# Varicocele and a Vascular Tumor in the Testis

**DOI:** 10.1155/2013/896142

**Published:** 2013-07-22

**Authors:** Christoph Kraft, Jan Janzen

**Affiliations:** ^1^Urologie, Baslerstrasse 66, 4600 Olten, Switzerland; ^2^Histopathologie, Worbstrasse 312, 3073 Gümligen bei Bern, Switzerland

## Abstract

A 50-year-old man presented a rare morphological constellation: a left-sided varicocele (stage 3) and a vascular rich Sertoli cell tumor.

## 1. Introduction

It is well known that varicocele leads to circulatory disturbances, testicular atrophy (shrinking), seminiferous tubule sclerosis, vessels degenerative changes, and abnormalities of Leydig, Sertoli, and germ cells [1, 2]. We have observed a high-grade varicocele in association with a testicular vascular tumor.

## 2. Case Presentation

**Figure 1:**
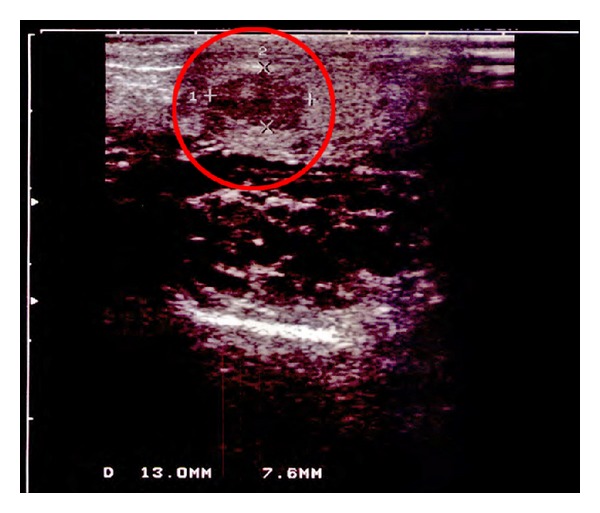
Tumor (red circle) and high-grade varicocele in sonography.

A 50-year-old man came to the urologist and presented a painful mass in the left scrotum. Clinically, a high-grade varicocele (stage 3) and a low testicular volume were found (Figure 1). Furthermore, a tumor in the upper pole area of the left testicle was observed. All tumor markers were negative; there were no symptoms of a renal cell carcinoma or nutcracker syndrome. Because of the low testicular volume, a left inguinal orchiectomy without intraoperative histopathological diagnosis (frozen section) was performed.

**Figure 2:**
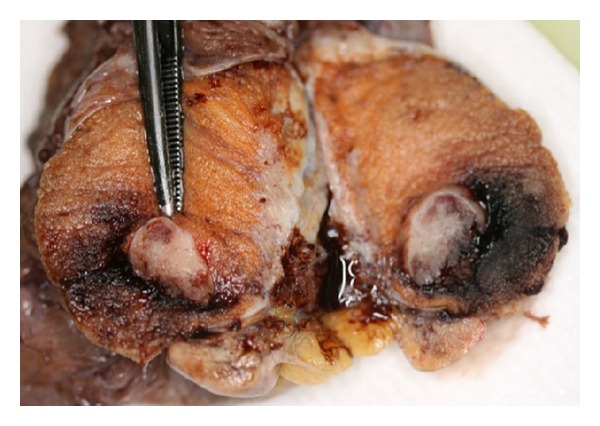
Macroscopic aspects of the benign testicular tumor.

**Figure 3:**
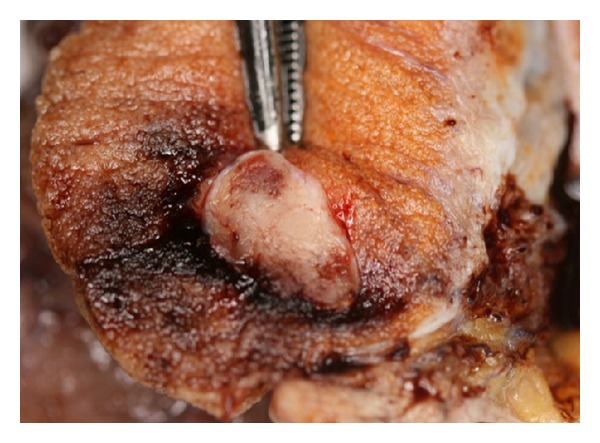
Vascular testicular tumor, measuring 12 mm in diameter.

Macroscopically, an abnormal dilation of the plexus pampiniformis and vessel mass measuring 50 mm in diameter were found. Left testicle measured 38 × 30 × 15 mm, and a 12 × 8 × 6 mm grey white solid tumor was seen (Figures 2 and 3). Rete testis, epididymis, and spermatic cord were inconspiciuous.

**Figure 4:**
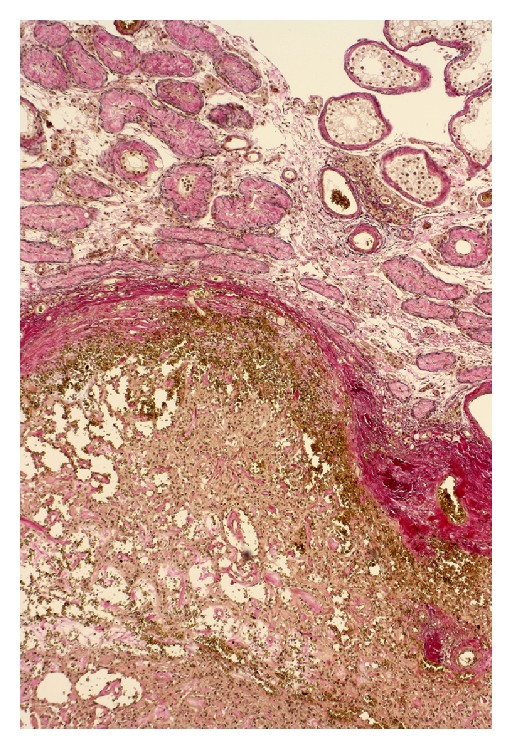
Microscopic aspects: benign vascular tumor and testicular parenchyma.

**Figure 5:**
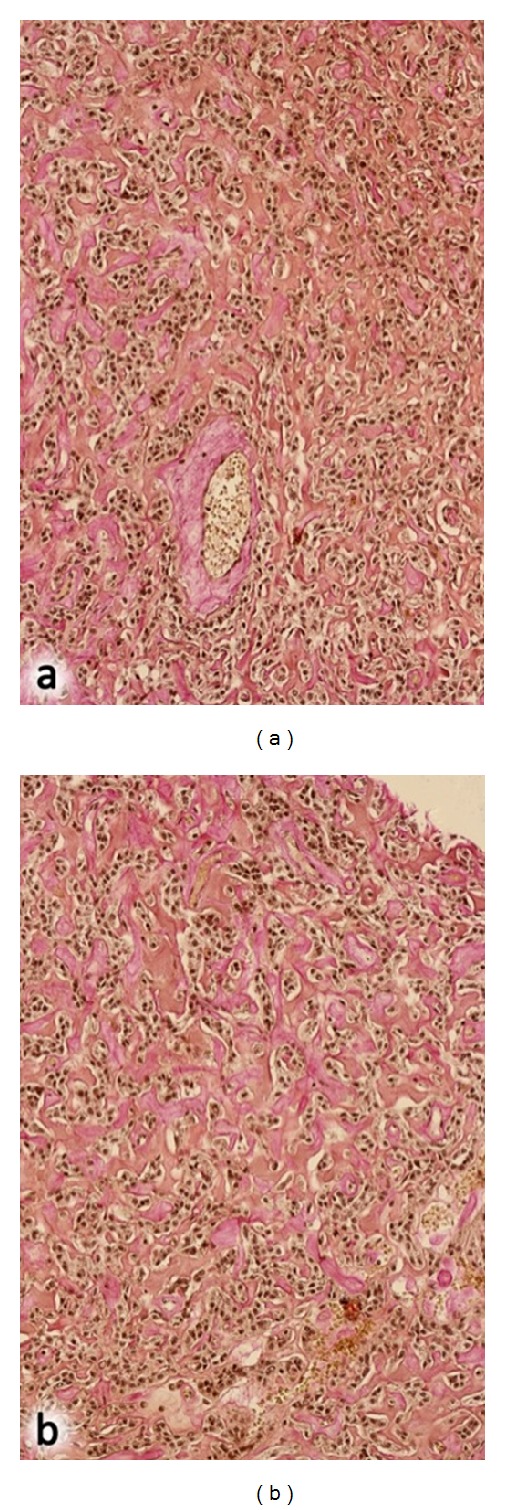
Microscopic aspects: Sertoli cells (a) and vascular components (b).

Microscopically, abnormal venous vessels in the plexus pampiniformis characterised by a severe leiomyomatous hyperplasia with incorporated nerve fibers were found. The lumen of the veins varied between 15 and 25 mm. Venous valves were absent. The testicular tumor had two microscopic patterns:

- vascular pattern in 70% of the tumor area: thick-wall vessels with CD34 expression and epithelioid cells,
- tubular pattern in 30% of the tumor area: hyalinised Sertoli cells with cytokeratin 18 expression (Figures 4 and 5).

Overall, a low proliferation rate (1 Mitosis/10 HPF), MIB1 (Ki67) in 3–5% of the tumor cells were detected; neither lymph nor hemangiosis carcinomatosa were observed. Adjacent testicular parenchyma showed a complete and arrested spermatogenesis, and a testicular intraepithelial neoplasia was excluded by immunohistochemistry (PLAP negative). We propose to classify this non-malignant tumor as a vascular rich Sertoli cell tumor of the testis.

## 3. Discussion

Morphologically, our case had two distinct entities—a high-grade varicocele and a vascular tumor of the testis. The benign tumor consists of vascular and tubular components with a clear dominance of vascular differentiated cells. To our knowledge, such a case has not been published yet in this particular clinical constellation. On one side, it could be that the existence of varicocele and benign tumor in the same testis is due to chance. On the other side, a long-lasting irregular blood circulation of the testis induced by varicocele (stage 3) could play an important role in the aetiopathogenesis [1]. Furthermore, the higher temperature of the testis induced by varicocele should be considered as a causative factor in the tumorigenesis [3]. 

The clinical take-home message of the case report is as follows: a high-grade varicocele can occur with a benign vascular tumor of the testis.
